# High‐Performance Organic Lithium Batteries with an Ether‐Based Electrolyte and 9,10‐Anthraquinone (AQ)/CMK‐3 Cathode

**DOI:** 10.1002/advs.201500018

**Published:** 2015-04-15

**Authors:** Kai Zhang, Chunyang Guo, Qing Zhao, Zhiqiang Niu, Jun Chen

**Affiliations:** ^1^ Key Laboratory of Advanced Energy Materials Chemistry (Ministry of Education)Collaborative Innovation Center of Chemical Science and EngineeringNankai UniversityTianjin300071China

**Keywords:** ether‐based electrolytes, high‐concentration Li salt, LiNO_3_ additives, mesoporous carbon, organic lithium batteries

## Abstract

Organic carbonyl electrode materials of lithium batteries have shown multifunctional molecule design and high capacity, but have the problems of poor cycling and low rate performance due to their high solubility in traditional carbonate‐based electrolytes and low conductivity. High‐performance organic lithium batteries with modified ether‐based electrolyte (2 m LiN(CF_3_SO_2_)_2_ in 1,3‐dioxolane/dimethoxyethane solvent with 1% LiNO_3_ additive (2m‐DD‐1%L)) and 9,10‐anthraquinone (AQ)/CMK‐3 (AQC) nanocomposite cathode are reported here. The electrochemical results manifest that 2m‐DD‐1%L electrolyte promotes the cycling performance due to the restraint of AQ dissolution in ether‐based electrolyte with high Li salt concentration and formation of a protection film on the surface of the anode. Additionally, the AQC nanocomposite improves the rate performance because of the nanoconfinement effect of CMK‐3 and the decrease of charge transfer impedance. In 2m‐DD‐1%L electrolyte, AQC nanocomposite delivers an initial discharge capacity of 205 mA h g^−1^ and a capacity of 174 mA h g^−1^ after 100 cycles at 0.2 C. Even at a high rate of 2 C, its capacity is 146 mA h g^−1^. This strategy is also used for other organic carbonyl compounds with quinone substructures and they maintain high stable capacities. This sheds light on the development of advanced organic lithium batteries with carbonyl electrode materials and ether‐based electrolytes.

## 1 Introduction

Organic electrode materials, especially organic carbonyl compounds, have recently captured attention because of their high capacity, molecule‐level controllable design, and resource sustainability.^1^, ^2^, ^3^, ^4^, ^5^, ^6^, ^7^, ^8^, ^9^, ^10^ However, organic carbonyl compounds are plagued by high solubility in traditional carbonate‐based electrolytes such as 1 m LiPF_6_ in ethylene carbonate and diethyl carbonate (EC/DEC) electrolyte, leading to rapid capacity decay.^11^, ^12^ Furthermore, the low conductivity of organic molecules limits their rate performance. The efforts to solve those issues are mainly divided into two categories: optimization of the electrolyte and modification of the electrode. For example, quasi‐solid‐state and all‐solid‐state electrolytes can effectively inhibit the dissolution of organic compounds.^13^, ^14^, ^15^, ^16^ Honma's group designed and synthesized a series of room‐temperature ionic liquid (RTIL) based quasi‐solid‐state electrolytes.^13^, ^14^ The selected RTIL made tetracyanoquinodimethane (TCNQ) delivers a discharge capacity of 170 mA h g^−1^ after 100 cycles with capacity retention of 78.8% at 0.2 C.^14^ Huang et al. prepared a poly(ethylene glycol) (PEG) based gel polymer electrolyte and a calix[4]quinone (C4Q) cathode, and the cell exhibited capacity retention of 89.8% after 100 cycles at 0.2 C.^15^ Although the conductivity of quasi‐solid‐state electrolytes can reach 10^−3^ S cm^−1^, the contact between electrolytes and electrode materials is still poor,^17^ leading to inferior rate performance.

The modification of the electrode mainly includes molecular design and enveloping organic materials in carbon framework. Increasing the polarity by using organic Li salts and enlarging the molecular weight through polymerization can reduce the solubility,^18^, ^19^, ^20^ but they introduce “dead mass” to decrease the specific capacity. When benzoquinone (BQ) forms Li salt (lithium 3,6‐dioxocyclohexa‐1,4‐diene‐1,4‐bis(olate), Li_2_C_6_H_2_O_4_), the theoretical specific capacity decreases from 496 to 353 mA h g^−1^.^11^ After polymerization, the theoretical specific capacity of polymer‐bound pyrene‐4,5,9,10‐tetraone (PPYT) is 147 mA h g^−1^ less than that of pyrene‐4,5,9,10‐tetraone (PTO, *C*_th_ = 409 mA h g^−1^).^19^ Furthermore, only a few studies focused on carbon–organic compound nanocomposite.^21^, ^22^, ^23^ Wang's group prepared disordered mesoporous carbon/9,10‐anthraquinone (AQ) nanocomposite with a discharge capacity of 97 mA h g^−1^ and capacity retention of 43.7% after 50 cycles.^21^ Li et al. synthesized CMK–3/2,2′‐bis(3‐hydroxy‐1,4‐naphthoquinone) (BHNQ) nanocomposite which delivered a first discharge capacity of 308.6 mA h g^−1^ and the capacity retention of 65.7% after 50 cycles at 0.1 C.^22^ Nevertheless, their cycling performance is not yet satisfied.

To maintain the high capacity with the improved cycling and rate performance, new strategies need to be proposed. It is noted that the problems of organic lithium batteries are similar to those of Li–S batteries including the intrinsic insulation of sulfur and high solubility of intermediate lithium polysulfides in organic electrolytes.^24^, ^25^, ^26^ The progress of Li–S batteries is attributed to the optimization of the electrolytes and the design of C–S composite cathodes.^17^, ^27^ For electrolytes, the replacement of carbonate‐based electrolytes by ether‐based electrolytes makes Li–S batteries achieve the reversible cycles.^28^, ^29^ There are very few reports about the use of ether‐based electrolytes for organic carbonyl compounds to obtain a high initial capacity,^30^, ^31^ but their cycling performance still has room to improve. Hu's group designed the “solvent‐in‐salt” (SIS) electrolyte with ultrahigh salt concentration and demonstrated that it could inhibit the dissolution of polysulfides and formation of Li dendrite.^32^ In addition, LiNO_3_ additive in ether‐based electrolyte can form protective film on the surface of anode to prevent the reaction between polysulfides and anode.^33^, ^34^ To the best of our knowledge, there is no report about ether‐based electrolyte containing high concentration Li salt and LiNO_3_ additive in the research of organic lithium batteries. For cathode materials, many carbon materials such as CMK‐3, carbon nanotubes (CNTs), graphene, and porous carbon spheres have been tried to coat or load sulfur,^35^, ^36^, ^37^ and the carbon materials not only provide a conductive pathway to fasten the electronic transport but also further constrain the dissolution of polysulfides.^24^ It is noted that CMK‐3 possesses the character of highly ordered porous structure, uniform pore size of ≈2 nm, large specific surface area, high conductivity, and wide applications such as hydrogen storage, supercapacitors, Li–S batteries, and Li‐ion batteries.^22^, ^35^, ^38^, ^39^ Especially, CMK‐3 has shown strong nanoconfinement effect for S cathode in the Li–S batteries.^35^ Therefore, combining the optimization of ether‐based electrolyte with CMK‐3‐based nanocomposite cathode is worth attempting.

**Figure 1:**
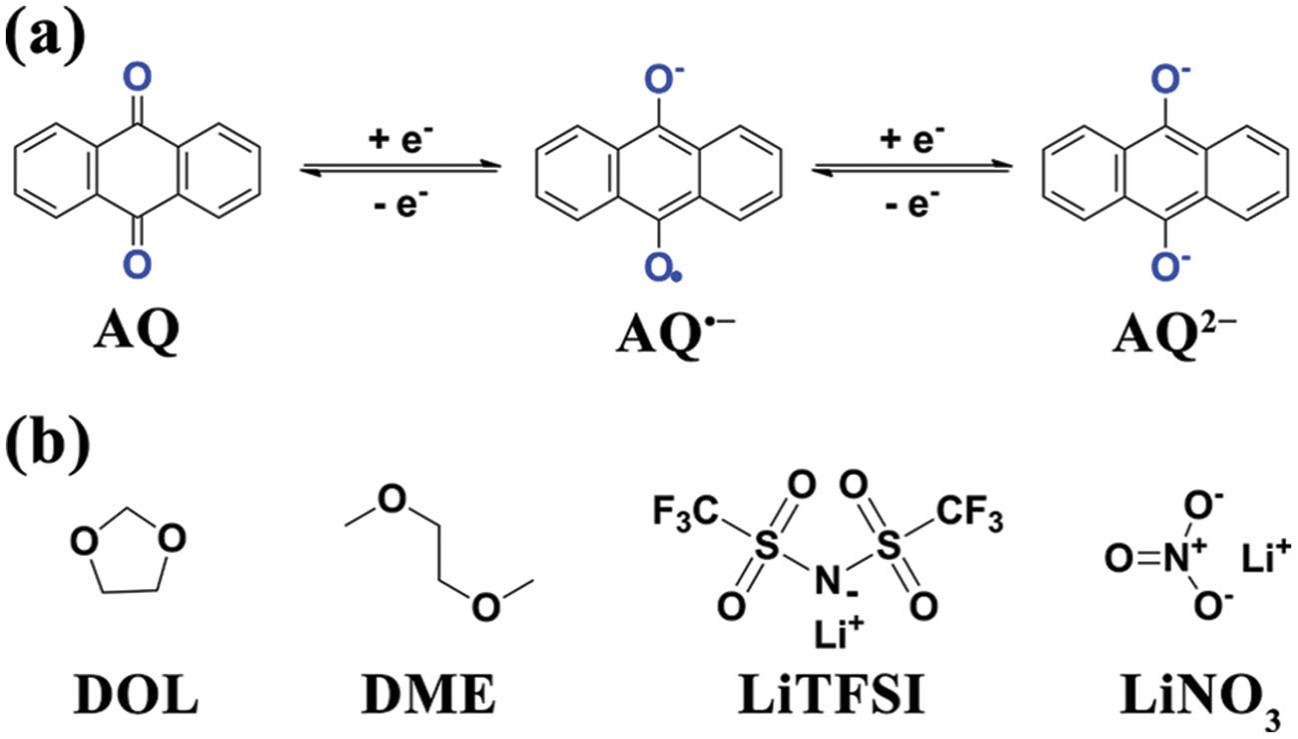
a) Structure and electrochemical redox reactions of AQ. b) Molecular structures of DOL, DME, LiTFSI, and LiNO_3_.

Herein, we report on high‐performance organic lithium batteries with modified ether‐based electrolyte and AQ/CMK‐3 (AQC) nanocomposite cathode. An organic molecule AQ with two‐electron reaction is selected as the cathode material (**Figure** **1**a). Meanwhile, the electrolyte consists of 1,3‐dioxolane/dimethoxyethane (DOL/DME) solvent, LiN(CF_3_SO_2_)_2_ (LiTFSI) salt, and LiNO_3_ additive (Figure 1b). Through optimizing the Li salt concentration and the additive amount, the 2 m LiTFSI in DOL/DME electrolyte with 1% LiNO_3_ additive (2m‐DD‐1%L) was chosen as an optimum electrolyte. Furthermore, CMK‐3 is used as a conductive matrix to envelope AQ for obtaining a nanoconfinement effect. The nanocomposite delivers an initial capacity of 205 mA h g^−1^ at 0.2 C and high capacity retention (84.9% after 100 cycles, 174 mA h g^−1^) as well as excellent rate capability (146 mA h g^−1^ at 2 C). To investigate the universal applicability of this strategy, 2m‐DD‐1%L and CMK‐3 are also extended to other organic carbonyl compounds with biphenyl quinone substructures, such as BHNQ, 4,4′‐dimethyl‐1,1′‐bi(cyclohexa‐3,6‐diene)‐2,2′,5,5′‐tetraone (DBT), 5,5′‐bibenzofuran‐4,4′,7,7′‐tetraone (BFT), and 2,2′‐binaphthyl‐1,1′,4,4′‐tetraone (BNT). The results also demonstrate that our proposed strategy is to further expand the development of organic lithium batteries with the combination of ether‐based electrolyte and carbon‐based nanocomposite cathode.

## 2 Results and Discussions

**Figure 2:**
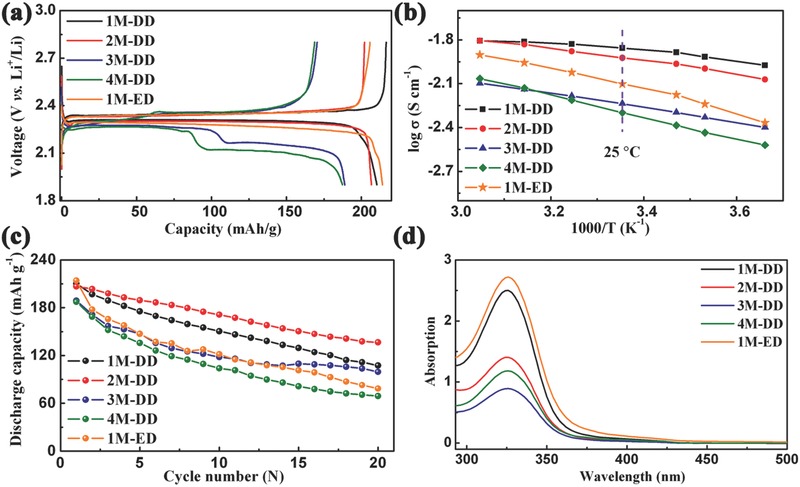
a) First discharge–charge curves of AQ with the five selected electrolytes (1m‐ED, 1m‐DD, 2m‐DD, 3m‐DD, and 4m‐DD; ED: ethylene carbonate/diethyl carbonate, DD: 1,3‐dioxolane/dimethoxyethane, *x* m: Li salt concentration) at 0.1 C. b) Arrhenius plots of the ionic conductivity as a function of 1000/T for the five electrolytes. c) Cycling performance of AQ with the five electrolytes at 0.1 C. d) The ultraviolet–visible spectra of the five electrolytes containing the same concentration of AQ (50 mg AQ in 2 mL of electrolyte), and before test the solutions are diluted with the corresponding electrolytes in a volume ratio of 1:20.

A series of ether‐based electrolytes were prepared by mixing LiTFSI salt and DOL/DME (1:1 by volume) solvent with salt concentrations of 1, 2, 3, and 4 m, which are labeled as 1m‐DD, 2m‐DD, 3m‐DD, and 4m‐DD, respectively. For comparison, 1 m LiPF_6_ in EC/DEC (1:1 by volume) denoted as 1m‐ED was also electrochemically tested. **Figure** **2**a shows the first discharge–charge curves of AQ with the above five electrolytes at 0.1 C. As to the traditional carbonate‐based electrolyte, 1m‐ED exhibits a discharge capacity of 214 mA h g^−1^ with a couple of redox plateaus at 2.35 and 2.28 V. For the ether‐based electrolytes, as the salt concentration increases, the discharge capacity gradually decreases. 1m‐DD and 2m‐DD deliver similar discharge capacities of 210 and 207 mA h g^−1^, respectively, with one discharge plateau at 2.30 V and one charge plateau at 2.35 V. Compared with 1m‐DD and 2m‐DD, 3m‐DD and 4m‐DD exhibit different charge–discharge curves with lower capacity and more plateaus. 3m‐DD presents two discharge plateaus at 2.28 and 2.15 V, and the discharge plateaus of 4m‐DD are located at 2.27 and 2.11 V. According to previous reports, AQ is first reduced to generate AQ^·−^, and then lose another e^−^ to form AQ^2−^.^11^, ^40^ The potential difference of each reaction step (Δ*V*) can be adjusted through changing the chemical and physical circumstance.^41^, ^42^ As shown in Figure 2b, the ionic conductivity at 25 °C reveals a decreasing order of 1m‐DD > 2m‐DD > 1m‐ED > 3m‐DD > 4m‐DD, illustrating that low ionic conductivity makes Δ*V* large to separate the two redox processes.

Figure 2c displays the cycling performance of AQ with the five electrolytes. Among them, 2m‐DD shows the highest discharge capacity and capacity retention after 20 cycles. To explain the reason for the difference of the cycling performance, the AQ dissolution experiments were tested. The color of all pristine electrolytes is transparent, and after soaking AQ for 4 d, the carbonate‐based and ether‐based electrolytes turn orange and light yellow, respectively (Figure S1, Supporting Information). After 30 d, the ether‐based electrolytes maintain the light yellow color, but the color of carbonate‐based electrolyte (1m‐ED) becomes brown. The AQ's solubility is also demonstrated by the analysis of ultraviolet–visible (UV–vis) spectra (Figure 2d). The five samples present the peaks of AQ at 325 nm in the UV–vis spectra, and the peak intensity follows an order of 1m‐ED > 1m‐DD > 2m‐DD > 3m‐DD > 4m‐DD, illustrating that the ether‐based electrolytes effectively decrease the AQ's solubility. 2m‐DD, 3m‐DD, and 4m‐DD show relative low peak intensity, and 2m‐DD also displays a relative high ionic conductivity of 11.9 mS cm^−1^. Taking into account the combination of ionic conductivity and AQ's solubility, 2m‐DD delivers the highest cycling performance. Thus, 2m‐DD is selected as the further optimized target.

**Figure 3:**
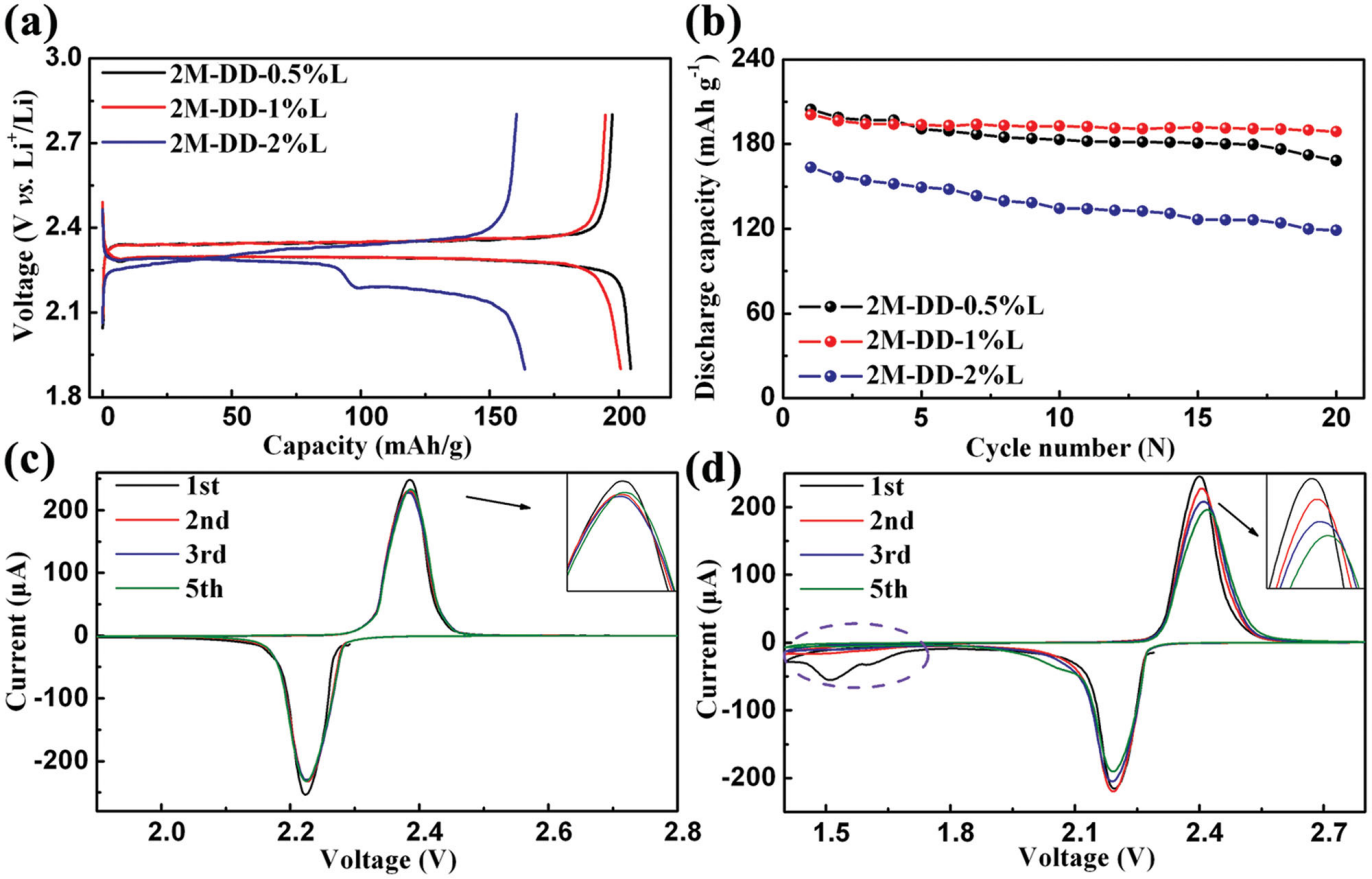
a) First discharge–charge curves and b) cycling performance of the AQ with different concentrations of LiNO_3_ additive at 0.1 C. Cyclic voltammograms of the AQ with 2m‐DD‐1%L electrolyte at a scan rate of 0.1 mV s^−1^ in the different range c) 1.9–2.8 V and d) 1.4–2.8 V.

According to previous researches about Li–S batteries,^34^, ^43^ using LiNO_3_ additive in ether‐based electrolytes can form a protective film on the Li anode surface to prevent the reactions between soluble polysulfides and metallic Li. Therefore, we added 0.5%, 1%, and 2% LiNO_3_ in 2m‐DD electrolyte named 2m‐DD‐0.5%L, 2m‐DD‐1%L, and 2m‐DD‐2%L, respectively. **Figure** **3**a shows the first discharge–charge curves of AQ with different concentrations of LiNO_3_ additive at 0.1 C. As LiNO_3_ concentration increases, the discharge capacity decreases. 2m‐DD‐0.5%L and 2m‐DD‐1%L exhibit similar discharge capacities of 204 and 201 mA h g^−1^, respectively, with a couple of charge/discharge plateaus. However, 2m‐DD‐2%L displays lower discharge capacity with two couples of charge/discharge plateaus. The difference of potential plateaus is also attributed to the low ionic conductivity of 2m‐DD‐2%L (Figure S2, Supporting Information).

Figure 3b presents the cycling performance of AQ with different concentrations of LiNO_3_ additive at 0.1 C. Table S1, Supporting Information, summarizes the 1st and 20th discharge capacities of 2m‐DD, 2m‐DD‐0.5%L, 2m‐DD‐1%L, and 2m‐DD‐2%L. Among them, 2m‐DD‐1%L delivers the best cycling performance with 94% capacity retention after 20 cycles. From the scanning electron microscopy (SEM) images (Figure S3, Supporting Information), it is obvious that when using LiNO_3_ additive, the surface of anode is smoother after 20 cycles compared with 2m‐DD sample. This demonstrates that LiNO_3_ additive can effectively form the protective film on the surface of anode to avoid the damage of anode. Raman analysis was also performed to investigate the anode surface after ten cycles when using 2m‐DD and 2m‐DD‐1%L as electrolytes. As shown in Figure S4 (Supporting Information), 2m‐DD sample shows the strong vibrations centered at around 1671 and 1599 cm^−1^, which are assigned to C=O stretching mode of AQ, while, 2m‐DD‐1%L sample does not exhibit obvious peak. This indicates that LiNO_3_ additive protects the anode. Since the low LiNO_3_ concentration (2m‐DD‐0.5%L) cannot form enough protective film and excessive LiNO_3_ concentration (2m‐DD‐2%L) may encumber the Li^+^ ion transfer (Figure S5, Supporting Information), 2m‐DD‐1%L is selected as the optimal electrolyte.

Figure 3c,d exhibits the cyclic voltammograms (CVs) of the AQ with 2m‐DD‐1%L electrolyte at a scan rate of 0.1 mV s^−1^ in different potential range. Between 1.9 and 2.8 V, there are a couple of well‐defined sharp redox peaks located at 2.38 V/2.23 V. The CV curves almost completely overlap after the first cycle. But a new irreversible peak is observed in the first reduction process between 1.4 and 2.8 V. The intensity of redox peaks gradually reduces during the cycles. Figure S6, Supporting Information, shows the cycling performance of AQ with 2m‐DD‐1%L electrolyte at 0.1 C between 1.5 and 2.8 V. After 20 cycles, the discharge capacity is only 127.2 mA h g^−1^, which is only 60.8% of the second discharge capacity. The poor cycling is attributed to setting a low cutoff voltage which induces the irreversible decomposition of the LiNO_3_ on the cathode like previous reports.^44^, ^45^ The decomposition of LiNO_3_ negatively impacts the cycling performance, so the voltage range is controlled between 1.9 and 2.8 V.

**Figure 4:**
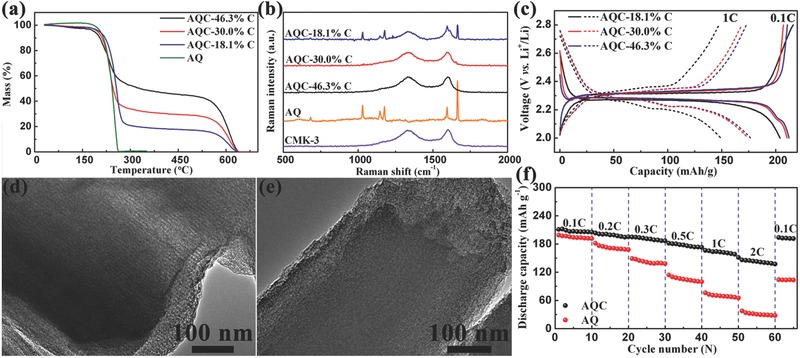
a) Thermogravimetric analysis curves of AQ and AQ/CMK‐3 (AQC) with different carbon contents. b) Raman spectra of CMK‐3, AQ, and AQC with different carbon contents. c) First discharge–charge curves of the AQC with different carbon contents at 0.1 C (line) and 1 C (dash). TEM images of d) CMK‐3 and e) AQC with 30 wt% CMK‐3. f) Discharge capacity of AQC with 30 wt% CMK‐3 and bare AQ cycled at various rates from 0.1 to 2 C.

Pure AQ possesses low electronic conductivity, which is adverse to its rate performance. To enhance its conductivity, AQ is impregnated into CMK‐3 to obtain AQC nanocomposite. The effect of carbon content on electrochemical performance is also investigated. **Figure** **4**a shows the thermogravimetric (TG) analysis curves of AQ and AQC with different carbon contents. The carbon contents of three AQC samples are analyzed to be 18.1, 30.0, and 46.3 wt%, respectively. The Raman spectra of CMK‐3, AQ, and three AQC samples are exhibited in Figure 4b. For AQC with 30.0 and 46.3 wt% CMK‐3, there is no obvious peak of AQ in Raman spectra, but AQC with 18.1 wt% CMK‐3 displays the clear characteristic peaks of AQ. This indicates that some AQ in AQC with 18.1 wt% CMK‐3 is not impregnated into the pores of CMK‐3. Figure S7, Supporting Information, shows the N_2_ adsorption and desorption isotherms and the corresponding pore‐size distributions of CMK‐3 and AQC samples. CMK‐3 has a large BET (Brunauer–Emmett–Teller) specific surface area of 1129.7 m^2^ g^−1^ with a pore volume of 1.29 cm^3^ g^−1^. The pore‐size distribution was characterized using the BJH (Barrett–Joyner–Halenda) method, and CMK‐3 possesses a uniform pore size of ≈2 nm. As the AQ content raises, the surface area and pore volume of AQC gradually decrease. AQC with 46.3 wt% CMK‐3 possesses a little pore volume of 0.18 cm^3^ g^−1^, while the pores of AQC with 30.0 and 18.1 wt% CMK‐3 are completely filled, which is consistent with the results of Raman spectra. Figure 4c displays the first discharge–charge curves of the AQC with different carbon contents at 0.1 and 1 C. The three samples deliver a couple of discharge/charge plateaus at 2.27 V/2.32 V at 0.1 C. AQC with 30.0 and 46.3 wt% CMK‐3 exhibit similar discharge capacities of 211 and 212 mA h g^−1^ based on the mass of AQ (same as below) at 0.1 C, respectively. While AQC with 18.1 wt% displays a lower discharge capacity of 204 mA h g^−1^, which is close to the capacity of pure AQ. At a high rate of 1 C, the discharge capacities of AQC with 18.1, 30.0, and 46.3 wt% CMK‐3 are 150, 175, and 177 mA h g^−1^, respectively. High CMK‐3 content improves the rate performance, but decreases the specific energy of the whole electrode. Thus, considering AQ loading content and capacity, 30.0 wt% CMK‐3 is optimum.

The transmission electron microscopy (TEM) images of CMK‐3 and AQC with 30.0 wt% CMK‐3 is shown in Figure 4d,e. CMK‐3 presents nanochanneled structure, while the mesoporous structure of CMK‐3 disappears in AQC with 30.0 wt% CMK‐3. This illustrates that AQ is impregnated into the nanochannels of CMK‐3 as called a nanoconfinement effect. The SEM image exhibits the smooth surface of AQC with 30.0 wt% CMK‐3, which also suggests that the pores are filled with AQ (Figure S8, Supporting Information). Figure 4f compares the rate performance of AQ and AQC with 30.0 wt% CMK‐3. As the current rate increases, the capacities of AQ decline much more rapidly than those of AQC with 30.0 wt% CMK‐3. At 0.1 C, AQ and AQC with 30.0 wt% CMK‐3 show initial discharge capacities of 199 and 211 mA h g^−1^, respectively. Even at high rates of 1 and 2 C, AQC with 30.0 wt% CMK‐3 still delivers high discharge capacities of 166 and 146 mA h g^−1^. After the current rate recovers to 0.1 C, the discharge capacity also turns back to 194 mA h g^−1^. However, the discharge capacity of pure AQ is only 37.6 mA h g^−1^ at 2 C, and it just regains 104 mA h g^−1^ when the rate turns back to 0.1 C. The excellent rate performance of AQC with 30.0 wt% CMK‐3 is attributed to the low charge transfer impedance (Figure S9, Supporting Information) and nanoconfinement effect of CMK‐3. Pure AQ is an electrical insulator and has microrod morphology (Figure S10, Supporting Information), which limits its utilization and Li^+^ ion diffusion. CMK‐3 not only improves the electronic conductivity but also manacle AQ into mesoporous structure,^22^, ^35^ leading to high specific capacity and rate performance.

**Figure 5:**
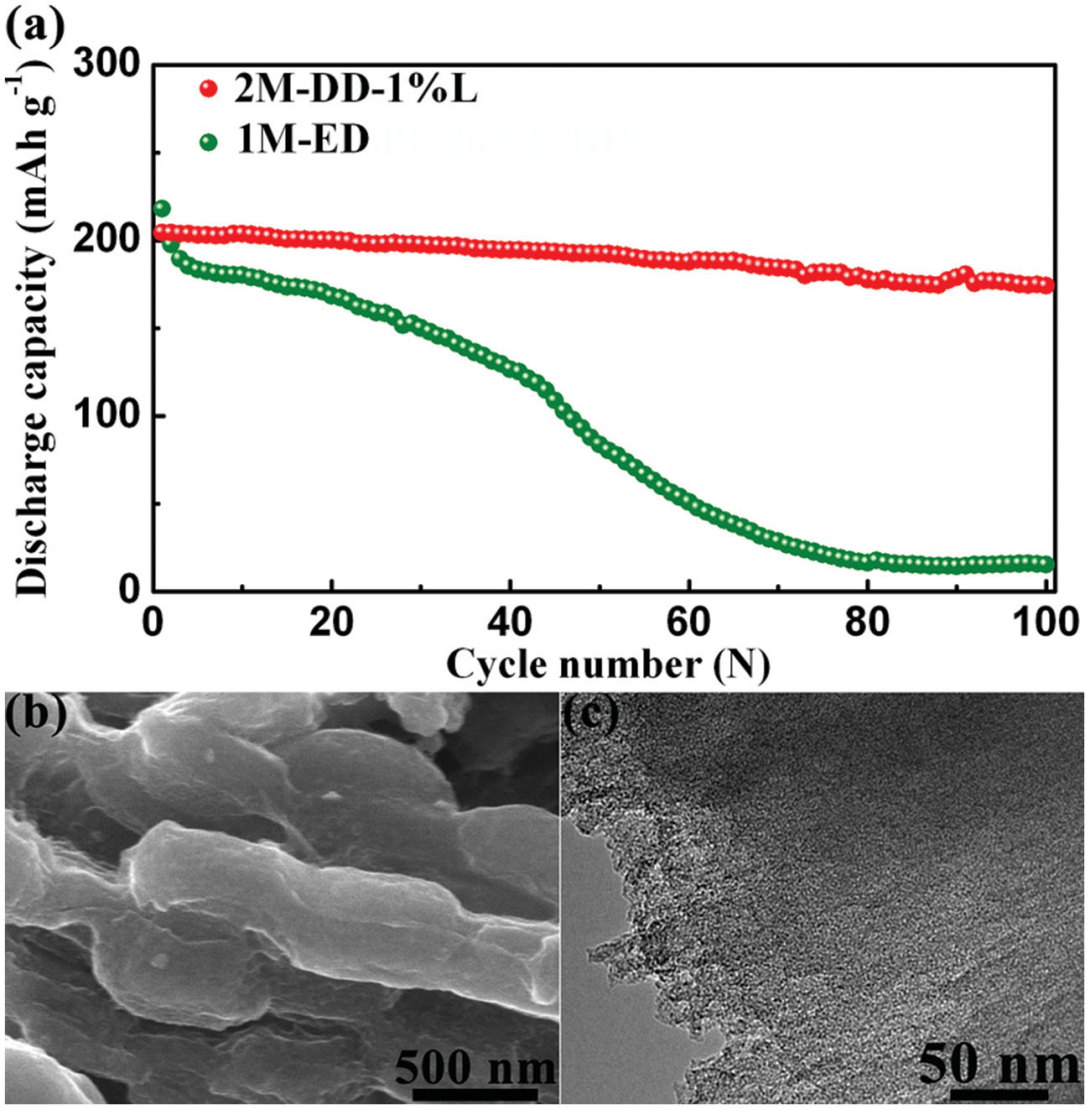
a) Cycling performances of AQC with 30 wt% CMK‐3 using ether‐based and carbonate‐based electrolytes at 0.2 C. b) SEM and c) TEM images of AQC with 30 wt% CMK‐3 after 100 cycles.

**Figure** **5**a compares the long‐term cycling performance of AQC with 30 wt% CMK‐3 using ether‐based and carbonate‐based electrolytes at 0.2 C. When 1m‐ED is used as the electrolyte, the discharge capacity rapidly decays, and only a capacity of 15.4 mA h g^−1^ can remain after 100 cycles. Using 2m‐DD‐1%L electrolyte, AQC with 30 wt% CMK‐3 delivers an initial discharge capacity of 205 mA h g^−1^, and after 100 cycles the discharge capacity still reach 174 mA h g^−1^, which corresponds to capacity retention of 84.9%. Table S2, Supporting Information, compares the cycling performance of AQ for lithium batteries in this study and those reported previously.^21^, ^30^, ^31^, ^46^, ^47^ It can be seen that our cycling performance is much higher than those reported. The SEM and TEM images of AQC with 30 wt% CMK‐3 using 2m‐DD‐1%L electrolyte after 100 cycles show an unchanged morphology and microstructure (Figure 5b,c). This indicates that AQ is well confined into the pores of CMK‐3. In comparison, we also used graphene as a conductive matrix. As shown in Figure S11, Supporting Information, AQ/graphene composite with 30 wt% graphene exhibits an initial capacity of 211 mA h g^−1^ at 0.2 C. After 20 cycles, the discharge capacity slightly decreases to 198 mA h g^−1^, which corresponds to capacity retention of 93.8%. As to AQC with 30 wt% CMK‐3, the discharge capacity and capacity retention are 200 mA h g^−1^ and 97.6% at after 20 cycles 0.2 C, which suggests that CMK‐3 has stronger nanoconfinement function than the case of graphene.

**Figure 6:**
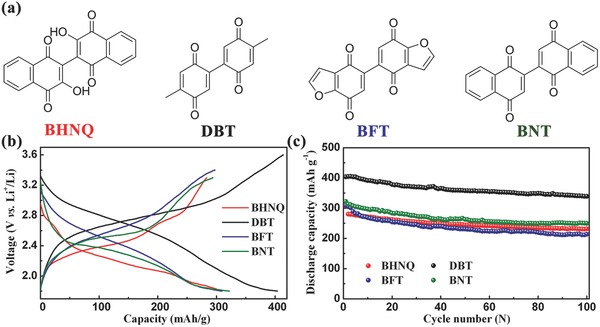
a) Molecular structures of the four selected organic carbonyl compounds. b) Discharge–charge curves and c) cycling performance of BHNQ, DBT, BFT, and BNT using 2m‐DD‐1%L electrolyte at 0.2 C.

To test the universality of our proposed strategy, 2m‐DD‐1%L electrolyte and CMK‐3 were also applied for other organic carbonyl compounds with biphenyl quinone substructures and higher theoretical specific capacities than that of AQ, such as BHNQ, DBT, BFT, and BNT. **Figure** **6**a shows the molecular structures of the four carbonyl compounds. They can achieve four‐electron reaction via four carbonyl groups to obtain high capacity, but they are also plagued by low electronic conductivity and high solubility in common organic electrolytes. Figure 6b exhibits the discharge–charge curves of BHNQ, DBT, BFT, and BNT at 0.2 C. BHNQ, DBT, BFT, and BNT deliver initial discharge capacities of 307, 404, 310, and 322 mA h g^−1^, respectively, which are 99.2%, 98.8%, 97.8%, and 98.5% of their theoretical specific capacities. Figure 6c displays the cycling performance of the four carbonyl compounds at 0.2 C, showing excellent cyclic stability. Especially for DBT, the capacity retention can reach 83.9% after 100 cycles. Furthermore, the cycling performance of BHNQ is also better than that of previous report^22^ as summarized in Table S2, Supporting Information. This manifests the wide applicability of combining 2m‐DD‐1%L electrolyte with CMK‐3‐based nanocomposites as an effective way for the improvement of electrode performance of organic carbonyl compounds with quinone substructures.

## 3 Conclusion

In conclusion, ether‐based electrolyte with high Li salt concentration and LiNO_3_ additive as well as AQ nanoconfined in CMK‐3 has been used to improve the cycling and rate performance of AQ. When using the carbonate‐based electrolyte and pure AQ as a cathode, the discharge capacity and capacity retention are only 79 mA h g^−1^ and 36.9% after 20 cycles at 0.1 C. But when the ether‐based electrolyte (2m‐DD‐1%L) and AQC cathode are used, the discharge capacity and capacity retention of AQC can reach 174 mA h g^−1^ and 84.9% after 100 cycles at 0.2 C. Furthermore, the AQC also delivers high discharge capacities at both low and high rates (211 mA h g^−1^ at 0.1 C, 205 mA h g^−1^ at 0.2 C, 166 mA h g^−1^ at 1 C, and 146 mA h g^−1^ at 2 C). The high cycling and rate performance of AQC is attributed to the following aspects: (1) ether‐based electrolytes with 2–4 m LiTFSI concentrations effectively decrease the solubility of organic compounds, (2) LiNO_3_ additive in the ether‐based electrolyte can form protection film on the surface of the anode, and (3) CMK‐3 not only reduces the charge transfer impedance but also has a nanoconfinement effect. This strategy is also demonstrated to be widely applicable for other organic carbonyl compounds with biphenyl quinone substructures, which could enlighten the wide application of this method for advanced organic lithium batteries.

## 4 Experimental Section

*Synthesis of Ether‐Based Electrolytes with LiNO_3_ Additive*: The ether‐based electrolytes were prepared by adding different contents of lithium bis(trifluoromethanesulfonyl)imide (LiN(SO_2_CF_3_)_2_, LiTFSI) (Shanghai Chengjie Co., China) into the mixed liquid of DOL (Alfa Aesar)/DME (J&K). The volume ratio of DOL to DME was 1:1. The LiTFSI concentrations are 1, 2, 3, and 4 m, respectively. The mixtures were stirred for 24 h at 25 °C under Ar atmosphere. The different contents of LiNO_3_ were added into 2 m LiTFSI DOL/DME electrolyte. The weight percents of LiNO_3_ were 0.5%, 1%, and 2%, respectively. The mixtures were also stirred for 24 h at 25 °C under Ar atmosphere.

*Synthesis of CMK‐3/9,10‐AQ Nanocomposites*: The different contents of AQ (J&K) were dissolved in dimethyl sulfoxide (DMSO). Then, CMK‐3 (Nanjing JiCang Co., China) was added to above solution. The weight ratios of AQ to CMK‐3 were 1:1, 2.3:1, and 4:1. After ultrasonic treatment for 30 min, the mixture was placed in oven under vacuum to remove the solvent at 100 °C.

*Material Characterizations*: NETZ SCH STA 449F thermal analyzer (Germany) was used to operate the TG analysis of AQ and AQC in the range from 30 to 700 °C at a rate of 5 °C min^‐1^ in air. The morphology and microstructure of the samples were observed by using SEM (JEOL, JSM‐7500F) and TEM (tecnai G2 F20). The ionic conductivity of the electrolytes was tested via AC impedance method using 0.1 m KCl (aq) as the reference material. The UV–vis spectra of the five electrolytes containing the same concentration of AQ (50 mg AQ in 2 mL of electrolyte) were recorded by a Jasco‐550 ultraviolet–visible (UV/vis) spectrophotometer. Before the UV–vis test, the solutions are diluted with the corresponding electrolytes in a volume ratio of 1:20. The surface area and pore volume of CMK‐3 and AQ/CMK‐3 composites were analyzed by N_2_ adsorption–desorption isotherms at 77 K on a BELSORP‐mini instrument. The Raman spectra were collected using a Thermo‐Fisher Scientific DXR Raman microscopy with a 532 nm wavelength. When testing the Raman spectra of Li anode, the Li was protected by thin Al_2_O_3_ glass, and was sealed in Ar atmosphere as shown in Figure S4a (Supporting Information).

*Electrochemical Tests*: The AQ electrode was prepared by mixing 60 wt% of AQ, 30 wt% of conductive carbon (super P), and 10 wt% of polyvinylidenefluoride (PVdF) binder in *N*‐methyl‐2‐pyrrolidone (NMP). For AQC cathode, the ratio of AQC:super P:PVdF was 8:1:1. The as‐prepared slurry was spread on Al foil and then dried in a vacuum oven at 90 °C for 12 h. Lithium metal was used as the anode and reference electrode, and Celgard 2320 was used as the separator. The CR2032 coin‐type cells were produced in an Ar‐filled glove box (Mikrouna Universal 2440/750). The as‐prepared cells were tested on a CT2001A cell test instrument (LAND Electronic Co.). CVs were carried out at a scan rate of 0.1 mV s^−1^ using a Parstat 263A electrochemical workstation (AMETEK Co.). Electrochemical impedance spectroscopy (EIS) measurements were performed on Parstat 2273 electrochemical workstation (AMETEK Co.). The AC perturb at ion signal was ±5 mV and the frequency range was from 100 mHz to 100 KHz.

## Supporting information

As a service to our authors and readers, this journal provides supporting information supplied by the authors. Such materials are peer reviewed and may be re‐organized for online delivery, but are not copy‐edited or typeset. Technical support issues arising from supporting information (other than missing files) should be addressed to the authors.

Supplementary
