# Supplementary material for: High‐Performance Organic Lithium Batteries with an Ether‐Based Electrolyte and 9,10‐Anthraquinone (AQ)/CMK‐3 Cathode
Source: Adv Sci (Weinh). 2015 Apr 15;2(5):1500018. doi: 10.1002/advs.201500018 (PMC5115363; doi:10.1002/advs.201500018)
Supplement: Supplementary file 1 — Supplementary [file ADVS-2-0k-s001.pdf]

Copyright WILEY-VCH Verlag GmbH & Co. KGaA, 69469 Weinheim, Germany, 2015.

ADVANCED SCIENCE
Open Access

Supporting Information

for *Adv. Sci.*, DOI: 10.1002/advs.201500018

High-Performance Organic Lithium Batteries with an Ether-Based Electrolyte and 9,10-Anthraquinone (AQ)/CMK-3 Cathode

Kai Zhang, Chunyang Guo, Qing Zhao, Zhiqiang Niu,* and Jun Chen*

WILEY-VCH

Copyright WILEY-VCH Verlag GmbH & Co. KGaA, 69469 Weinheim, Germany, 2013.

Supporting Information

High-performance organic Li-ion batteries with ether-based electrolyte and 9,10-anthraquinone(AQ)/CMK-3 cathode

Kai Zhang, Chunyang Guo, Qing Zhao, Zhiqiang Niu* and Jun Chen*

List of short-name

1. ethylene carbonate/diethyl carbonate (EC/DEC)
2. lithium bis(trifluoromethanesulfonyl)imide (LiTFSI)
3. 1,3-dioxolane/dimethoxyethane (DOL/DME)
4. 1M $LiPF_6$ in EC/DEC (1M-ED)
5. *x*M LiTFSI in DOL/DME (*x*M-DD, *x*=1, 2, 3, and 4)
6. 2M-DD with *y*% $LiNO_3$ (2M-DD-*y*%L, *y*=0.5, 1, and 2)
7. 9,10-anthraquinone (AQ)
8. AQ/CMK-3 (AQC)

Figures S1-S11
Table S1-S2

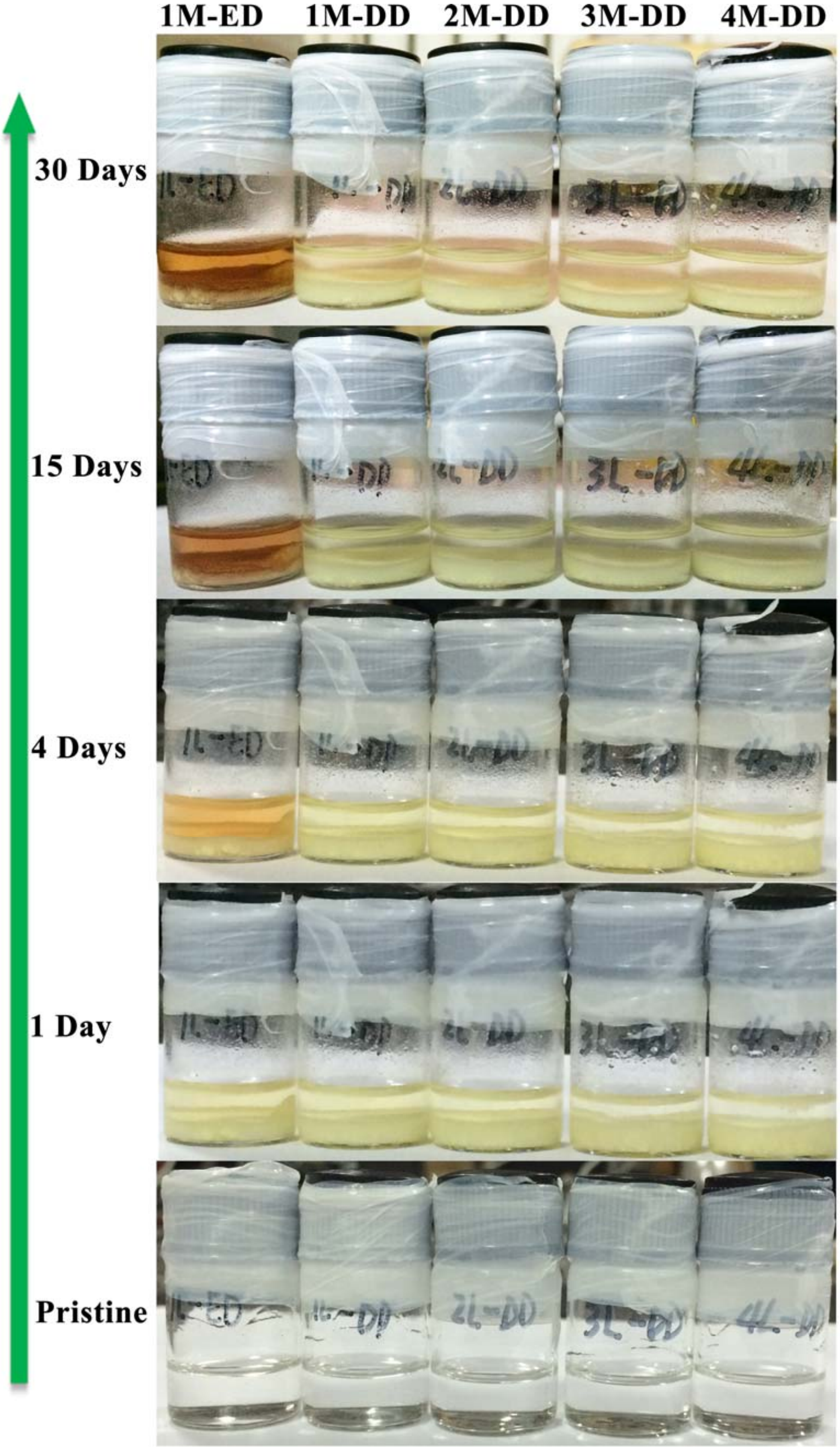

Figure S1. Photographes of the pure 1M-ED, 1M-DD, 2M-DD, 3M-DD, and 4M-DD electrolyte and the five electrolytes containing the AQ (50 mg AQ in 2 mL of electrolyte) for 1, 4, 15, and 30 days.

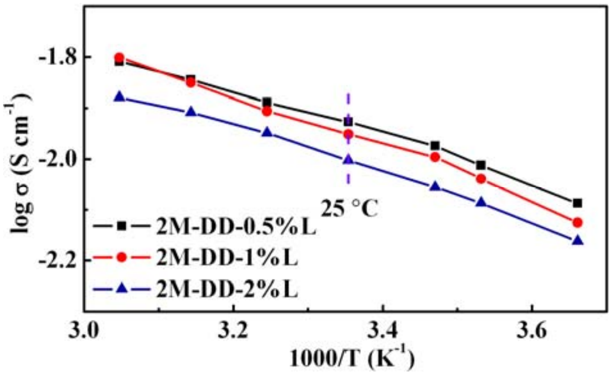

Figure S2. Arrhenius plots of the ionic conductivity as a function of 1000/T for the 2M-DD with different concentrations of $LiNO_3$ additive.

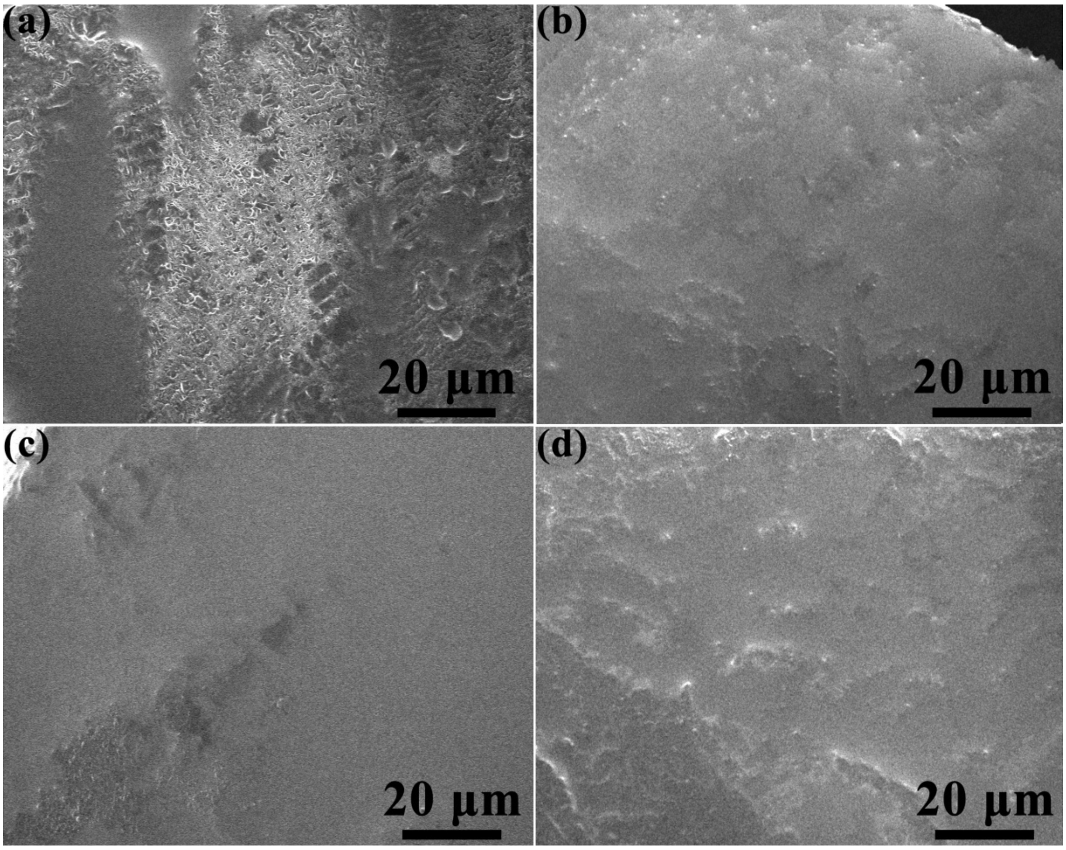

Figure S3. Scanning electron microscopy (SEM) images of metallic lithium anodes cycled after 20 cycles using different electrolytes (a. 2M-DD electrolyte, b. 2M-DD-0.5%L electrolyte, c. 2M-DD-1%L electrolyte, and d. 2M-DD-2%L electrolyte).

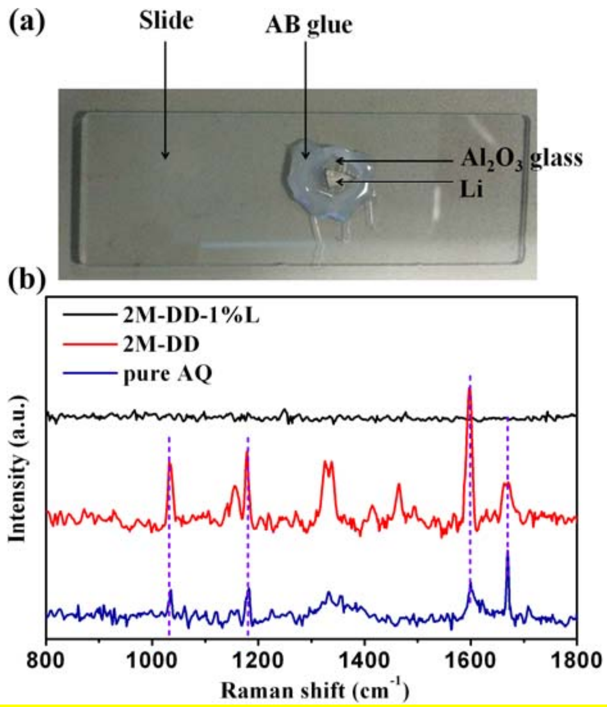

Figure S4. (a) Photograph of the Raman test instrument of Li anode. Li anode was protected by thin Al_2O_3 glass, and was sealed in Ar. (b) Raman spectra of pure AQ (blue) and Li anode suface after 10 cycles for 2M-DD (red) and 2M-DD-1%L (black) electrolytes.

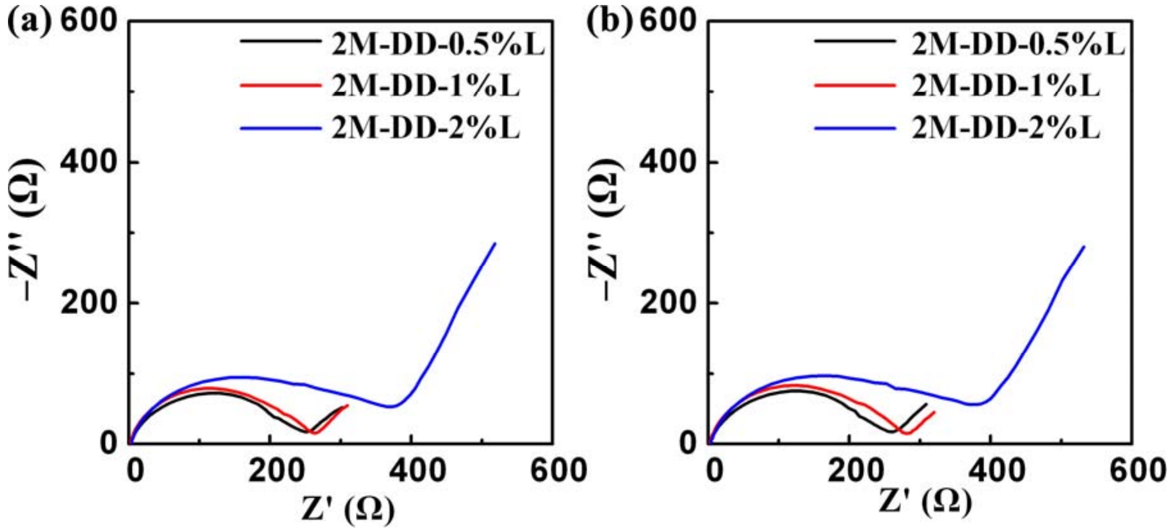

Figure S5. Electrochemical impedance spectroscopy (EIS) of the cells with different concentrations of $LiNO_3$ additive tested at 25% (a) and 75% (b) depth of discharge after 5 cycles.

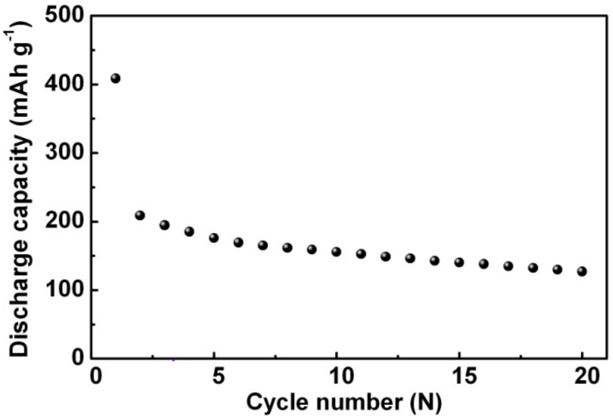

Figure S6. Cycling performance of the AQ with 2M-DD-1%L electrolyte at 0.1C between 1.5 and 2.8 V.

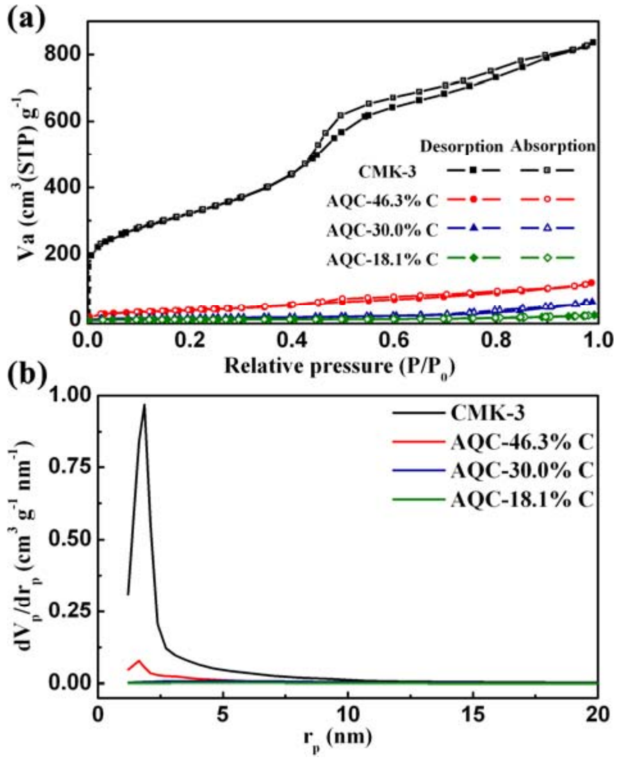

Figure S7. (a) N_2 adsorption and desorption isotherms at 77 K and (b) the corresponding pore-size distributions of CMK-3 and AQC with different carbon contents.

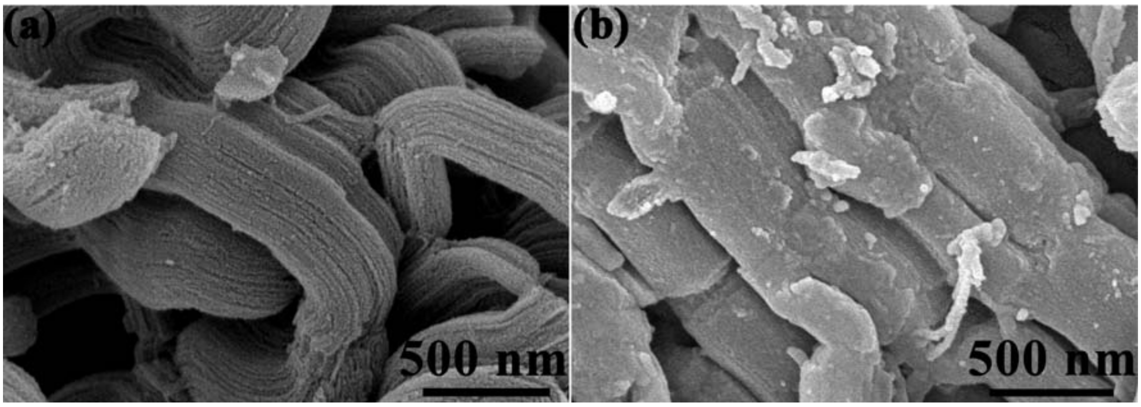

Figure S8. SEM images of CMK-3 (a) and AQC with 30 wt% carbon (b).

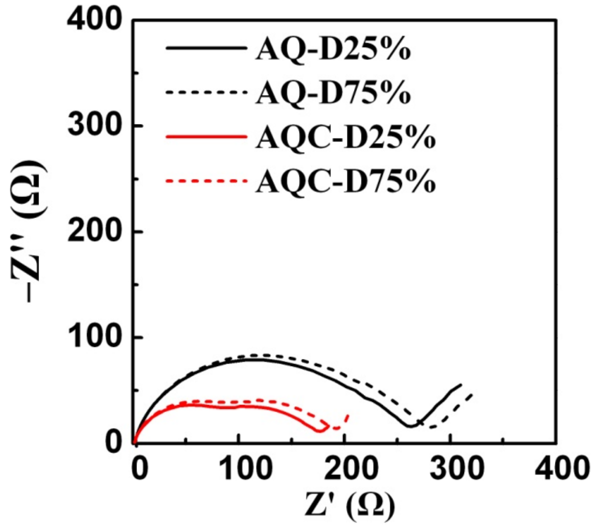

Figure S9. EIS of the cells with 2M1L electrolyte using AQ and AQC-30 wt% C as cathode tested at 25% (D25%) and 75% (D75%) depth of discharge after 5 cycles.

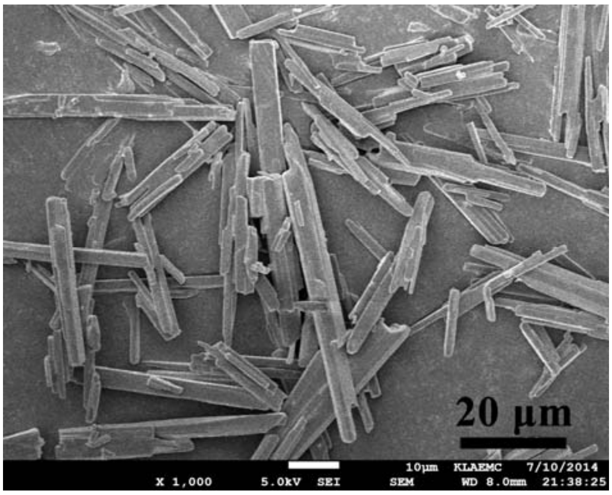

Figure S10. SEM image of commercial AQ powder.

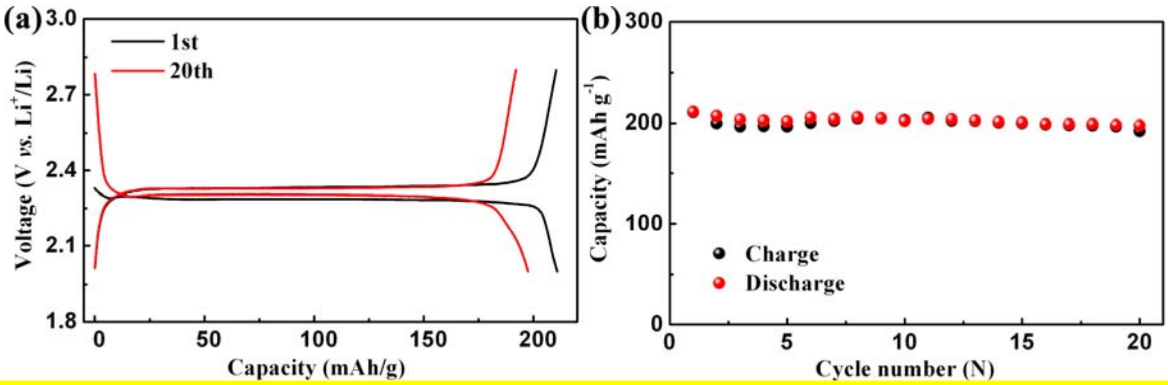

Figure S11. Discharge-charge curves (a) and cycling performance of the AQ/graphene composite at 0.2 C. The graphene was prepared like the previous report.[1]

Table S1. Comparison of the 1st and 20th discharge capacity of the 2M-DD, 2M-DD-0.5%L, 2M-DD-1%L, and 2M-DD-2%L.

Sample	1st discharge capacity (mAh g^{-1})	20th discharge capacity (mAh g^{-1})
**2M-DD**	207	137
**2M-DD-0.5%L**	204	168
**2M-DD-1%L**	201	189
**2M-DD-2%L**	164	119

Table S2. Comparison of the cycling performance in this study with the previous reports about AQ and BHNQ for lithium batteries.

**Molecular**	**Cycle number**	**100th discharge capacity (mAh g^{-1})**	**Capacity retention**	**Ref.**
**AQ**	100	174	84.9%	This work
**AQ**	50	100	45.0%	[2]
**AQ**	50	71	31.4%	[3]
**AQ**	50	62	29.0%	[4]
**AQ**	100	49	22.6%	[5]
**AQ**	50	100	39.8%	[6]
**BHNQ**	100	232	75.6%	This work
**BHNQ**	50	203	65.7%	[7]

References

[1] K. Zhang, L. Wang, Z. Hu, F. Cheng, J. Chen, *Sci. Rep.* **2014**, *4*, 6467.
[2] L. Zhao, W. Wang, A. Wang, Z. Yu, S. Chen, Y. Yang, *J. Electrochem. Soc.* **2011**, *158*, A991.
[3] L. Qiu, Z. Shao, M. Liu, J. Wang, P. Li, M. Zhao, *Carbohydr. Polym.* **2014**, *102*, 986.
[4] L. Xie, L. Zhao, J. Wan, Z. Shao, F. Wang, S. Lv, *J. Electrochem. Soc.* **2012**, *159*, A499.
[5] M. Yao, S.-I. Yamazaki, H. Senoh, T. Sakai, T. Kiyobayashi, *Mater. Sci. Eng. B* **2012**, *177*, 483.
[6] Z. Song, H. Zhan, Y. Zhou, *Chem. Commun.* **2009**, 448.
[7] H. Li, W. Duan, Q. Zhao, F. Cheng, J. Liang, J. Chen, *Inorg. Chem. Front.* **2014**, *1*, 193.
